# Predictors and Consequences of Inappropriate Thyroid Ultrasound in Hypothyroidism

**DOI:** 10.7759/cureus.17304

**Published:** 2021-08-19

**Authors:** Kaitlyn V Barrett, Amanda G Kennedy, Allen B Repp, Bradley J Tompkins, Matthew P Gilbert

**Affiliations:** 1 Larner College of Medicine, University of Vermont, Burlington, USA; 2 Department of Medicine, University of Vermont Medical Center, Burlington, USA

**Keywords:** ultrasound thyroid, medical waste, thyroid scan, inappropriate testing, non-invasive imaging

## Abstract

Introduction

In 2012, the American Board of Internal Medicine Foundation established the Choosing Wisely® initiative, partnering with specialist societies to promote evidence-based care. Under this program, the Endocrine Society recommends against ordering thyroid ultrasounds in individuals with subclinical or overt hypothyroidism and a normal neck exam. We sought to understand the prevalence, predictors, and consequences of thyroid ultrasound performed at our academic medical center that are not in compliance with this recommendation.

Methods

We conducted a retrospective cohort study of electronic health record data from January 1, 2016 to July 31, 2018. Data were extracted from records of all patients who underwent thyroid ultrasonography. Ultrasounds were considered inappropriate if they were ordered based on hypothyroidism, without other clear indications.

Results

A total of 2,021 patients underwent thyroid ultrasonography, of which 572 (28.3%) were diagnosed with hypothyroidism. Among the patients with hypothyroidism, 40 were identified as having received an inappropriate ultrasound (7.0%). Of those patients who received inappropriate ultrasounds, 42.5% had subsequent medical encounters, with a mean charge of $851 (standard deviation = $271) per patient. Using a multivariable model, the odds of receiving an inappropriate ultrasound were significantly higher for patients younger than 50 years of age (odds ratio: 2.37, 95% confidence interval: 1.01-5.58).

Conclusion

Seven percent of thyroid ultrasounds were inappropriately ordered in a cohort with hypothyroidism. Patients aged <50 years were at an increased risk of inappropriate ultrasound. Sequelae of inappropriate ultrasound included further medical encounters and financial burdens. Systems to reduce the inappropriate use of thyroid ultrasound may lessen the consequences of unnecessary medical imaging.

## Introduction

Hypothyroidism affects 3.7% of the population [1]. Hypothyroidism is diagnosed based on patient history, physical examination, and thyroid function tests [2]. High-resolution ultrasonography is a sensitive imaging modality for evaluating thyroid structure; however, it often results in clinically insignificant findings unrelated to abnormal thyroid function [3]. Thyroid ultrasound is recommended for patients who are at risk for thyroid malignancy, have palpable thyroid nodules or goiter, or have neck lymphadenopathy [4]. Thyroid ultrasound is not recommended as a screening test for patients with normal thyroid on palpation [4]. Per the Choosing Wisely® initiative of the American Board of Internal Medicine Foundation, the Endocrine Society recommends against ordering thyroid ultrasounds in individuals with subclinical or overt hypothyroidism and a normal neck exam [5].

Studies suggest variability in meeting this recommendation [5-7]. In one study of family physicians, 18.8% of thyroid ultrasound requests did not meet practice guideline indications, with 5.6% of thyroid ultrasounds deemed unnecessary based on guidelines of The Royal College of Radiologists [6]. Additionally, Liel and Fraenkel found that 93% of thyroid ultrasound orders in first-time endocrinology clinic referrals were considered inappropriate, and that 5.2% of patients with hypothyroidism were inappropriately evaluated with ultrasound [7].

The potential consequences of inappropriate thyroid ultrasound include unnecessary care and additional costs. Overuse of thyroid ultrasound can lead to an increased diagnosis of low-risk or insignificant thyroid cancer [8]. Of patients who undergo fine-needle aspiration (FNA) biopsy for incidental thyroid nodules, 22% to 41% proceed to surgery due to inconclusive cytological results. Retrospective evaluation of these patients found that after surgery, as many as 76% had benign results [9]. Even in patients who are ultimately found to have benign biopsies, follow-up imaging and repeat FNAs create a significant financial and emotional burden on the patient. In a study from a US academic medical center, 47.5% of patients with benign biopsy results had medical costs estimated at $1,099 per patient [10].

Few studies have evaluated the prevalence, predictors, and consequences of ordering inappropriate thyroid ultrasounds. The purpose of this study was to understand the prevalence of inappropriate thyroid ultrasounds that occur at an academic medical center, including the predictors of inappropriate thyroid ultrasounds as well as the consequences, from both clinical and financial perspectives.

## Materials and methods

This was a retrospective cohort study of electronic health record data (Epic) at the University of Vermont Medical Center (UVMMC). UVMMC is an academic institution and a 562-bed regional referral center for approximately 1 million people in Vermont and northern New York. Data were extracted from all patients who underwent thyroid ultrasound (procedure code US309) performed between January 1, 2016 and July 31, 2018. There were no cases of exclusions. This study was approved by the University of Vermont Committees on Human Research (# 19-0215).

Data related to the thyroid ultrasound order included ordering provider name, ordering provider specialty, clinical indication free text, and date of ultrasound. Patient data included a diagnosis of hypothyroidism (ICD-10 E03.9) if present, first date of hypothyroidism in the problem list of the encounter diagnosis, thyroid-stimulating hormone (TSH) and free T4 date and result immediately prior to the ultrasound, history of any cancer, history of endocrine cancer, history of neck radiation, and family history of thyroid cancer.

Patient records with “hypothyroidism” listed as a diagnosis or clinical indication for ultrasound were flagged as having received a potentially inappropriate thyroid ultrasound. Flagged charts were subsequently reviewed manually. Manual chart review included evaluation of the last relevant clinical note to assess documentation of abnormal neck examination or other rationale for thyroid ultrasound.

Upon determination of inappropriate thyroid ultrasound, records were reviewed for a one-year period following the initial thyroid ultrasound to assess for the following procedures: repeat ultrasound, biopsy (CPT codes 76941, 10022), endocrinology or otolaryngology referral, and surgical thyroidectomy (60240) or lobectomy (60220). Data were obtained on procedure charges in U.S. dollars for all cases in which an inappropriate thyroid ultrasound was ordered.

Univariate logistic regression was performed on the following predictors: patient age, sex, body mass index (BMI), current smoking status, radiation history, endocrine cancer history, general cancer history, family history of thyroid cancer, and the medical specialty of the provider who ordered the ultrasound. Patient age was categorized as less than or greater than 50 years. Predictors with a P-value ≤ 0.20 in the univariate model were then included in a multivariable logistic regression model. All statistical analyses were performed using Stata version 15.1 (Stata Corp LLC, College Station, TX, USA).

## Results

The study population consisted of 2,021 patients who underwent thyroid ultrasound at UVMMC during the 30-month study period. Of the entire cohort, 572 (28.3%) patients were diagnosed with hypothyroidism. Of those patients with hypothyroidism, 40 (7%) were identified as having received an inappropriate thyroid ultrasound. In the inappropriately ordered ultrasound group, 82.5% of patients were female with a mean age of 48.4 ± 12.7 years and mean BMI of 29.9 ± 7.5 (Table 1). A total of 12.5% were current smokers, compared to 5.3% in the appropriate ultrasound group. No patients in the inappropriate ultrasound group had a history of radiation, thyroid, or endocrine cancer.

**Table 1:** Demographics and health history of patients with hypothyroidism (n = 572) *BMI was available for 249 patients (N = 236 for appropriate, N = 13 for inappropriate ultrasound). **Smoking status was available for 379 patients (N = 355 for appropriate, N = 24 for inappropriate ultrasounds)

Characteristic	All Hypothyroid Patients (n = 572)		Appropriate Ultrasounds (n = 532)		Inappropriate Ultrasounds (n = 40)	
Female Sex, n (%)	480	(83.9)	447	(84.0)	33	(82.5)
Age, mean (SD)	52.9	(15.4)	53.2	(15.5)	48.4	(12.7)
Age <50 years, n (%)	234	(40.9)	212	(39.8)	22	(55.0)
BMI*, mean (SD)	29.9	(7.4)	29.9	(7.4)	29.9	(7.5)
Current Smoker**, # (%)	22	(5.8)	19	(5.3)	3	(12.5)
Ever Smoked**, # (%)	126	(33.2)	118	(33.2)	8	(33.3)
History of Any Cancer, # (%)	128	(22.4)	127	(23.9)	1	(2.5)
Family History of Thyroid Cancer, # (%)	27	(4.7)	27	(5.1)	0	(0.0)
History of Radiation, # (%)	15	(2.6)	15	(2.8)	0	(0.0)

The multivariable logistic regression model included 379 patients who had complete data for the two predictors; age <50 years and current smoking status. Patients aged <50 years were more likely to receive an inappropriate thyroid ultrasound (odds ratio [OR] = 2.37, 95% confidence interval [CI] = 1.01-5.58, p = 0.048). Current smokers also tended to be more likely to receive an inappropriate ultrasound (OR = 2.29, 95% CI 0.62-8.47, p = 0.216); however, this trend was not statistically significant.

Of the 40 patients who received inappropriate ultrasounds, 42.5% had subsequent medical encounters in the year following the ultrasound, with a mean charge of $851 (standard deviation = $271) per patient (Table 2). Some patients had more than one type of medical encounter. The most common type of encounter following an inappropriate ultrasound in hypothyroidism was an endocrinology or otolaryngology consult, with a mean charge of $855 per patient (standard deviation = $289).

**Table 2:** Post-ultrasound related medical encounters among hypothyroidism patients (n = 572) ENT: Ear, nose, and throat

	All Hypothyroid Patients (n = 572)		Appropriate Ultrasounds (n = 532)		Inappropriate Ultrasounds (n = 40)	
Medical Encounter	#	%	#	%	#	%
Endocrine or ENT consult	378	(66.1)	363	(68.2)	15	(37.5)
Repeat ultrasound	104	(18.2)	101	(19.0)	3	(7.5)
Biopsy	103	(18.0)	102	(19.2)	1	(2.5)
Thyroidectomy	22	(3.8)	21	(3.9)	1	(2.5)
Lobectomy	22	(3.8)	22	(4.1)	0	(0.0)

## Discussion

At our academic medical center, 7% of thyroid ultrasounds performed during the study period in patients with hypothyroidism were deemed inappropriate. Age <50 years was associated with an increased risk of receiving an inappropriate thyroid ultrasound. Sequelae of inappropriate thyroid ultrasounds included further medical encounters, repeat ultrasound, biopsy, thyroid surgery, and subspecialty consultation. We estimated that in total, $34,040 of excess charges were incurred due to inappropriate thyroid ultrasounds performed in the patients with hypothyroidism over the study period (that is, 40 patients × $851 of mean excess charges per patient). In terms of overall health care expenditure, this number is surprisingly low. Although the overall measured financial charges were not as high as expected, one must consider the existence of a number of unmeasured costs; in addition to the cost of the procedure and subsequent visits, there are costs associated with missed work, patient and clinician time, productivity loss, and the emotional cost of worry and pain for the patient.

Our findings are similar to those of previous studies; a single-center analysis performed by Liel and Fraenkel found that 5.2% of thyroid ultrasounds were obtained inappropriately for hypothyroidism, and Davenport et al. found that 6.6% of thyroid ultrasounds over a one year period were inappropriately ordered under the indication of hypo/hyperthyroidism [7,11]. While there are other important inappropriate indications for thyroid ultrasound (e.g., euthyroid patients with positive thyrotropin receptor antibodies and family history of hypothyroidism), to our knowledge, this is the only study that specifically examines biochemically hypothyroid patients who received inappropriate thyroid ultrasound according to the Choose Wisely® guidelines.

There were some limitations to our study. The study was conducted at a single center, and retrospective chart reviews and analyses were exploratory. The follow-up period was short, lasting only for one year. In addition, many of the ultrasounds were deemed inappropriate if hypothyroidism was listed as the diagnosis and there was no documentation to provide other reasons for the ultrasound. Therefore, although a patient may have had an appropriate reason for ultrasound, if it was not indicated on the radiology ultrasound request form, the ultrasound was deemed inappropriate. This multivariable model only explains a small percentage of the variation between groups receiving appropriate vs. inappropriate ultrasounds; thus, there are likely additional unexplored factors that contribute to ordering inappropriate thyroid ultrasounds.

## Conclusions

This study provides insights into the predictors of receiving an inappropriate thyroid ultrasound and identifies opportunities for quality improvement initiatives to reduce the number of unnecessary ultrasounds ordered for hypothyroidism at our institution. One opportunity for quality improvement is education concerning ordering thyroid ultrasounds in patients less than 50 years of age. Possible mechanisms for implementing this improvement include providing information for ordering providers, either embedded in the medical record, or in the form of a lecture or educational email. Other opportunities include automated “hard-stops” within order forms which would prevent inappropriate ultrasound requests. Moving forward, systems to reduce the inappropriate use of thyroid ultrasound may lessen the consequences of unnecessary medical imaging and the cost of such procedures.
